# Cases of acute coronary syndrome and presumed cardiac death prior to arrival at an urban tertiary care hospital in Pakistan during the COVID-19 pandemic

**DOI:** 10.1371/journal.pone.0263607

**Published:** 2022-02-03

**Authors:** Sana Sheikh, Wil Van Cleve, Vinod Kumar, Ghazal Peerwani, Saba Aijaz, Asad Pathan

**Affiliations:** 1 Department of Clinical Research Cardiology, Tabba Heart Institute, Karachi, Pakistan; 2 Department of Anesthesiology and Pain Medicine, Institute for Health Metrics and Evaluation, University of Washington, Seattle, WA, United States of America; 3 Department of Emergency, Tabba Heart Institute, Karachi, Pakistan; Fondazione IRCCS Policlinico San Matteo, ITALY

## Abstract

**Background:**

A reduction in overall acute coronary syndrome (ACS) cases, increases in the severity of ACS presentation, and increased rates of out-of-hospital cardiac arrest (OHCA) have been reported from multiple countries during the COVID-19 pandemic. The attributed factors include COVID-19 infection, fear of COVID-19 and resultant avoidance of health care facilities, and restrictions on mobility. Pakistan, a country with a high burden of cardiovascular disease (CVD) and challenges related to health care access, will be expected to demonstrate these same findings. Therefore, we compared ACS hospitalization, ACS severity, and patients who have already died (dead on arrival, or DOA) due to presumed OHCA at a tertiary cardiac hospital during pre-pandemic and intra-pandemic periods in Pakistan.

**Methods:**

Standardized data elements were extracted from the charts of patients with ACS, and telephonic verbal autopsies (VA) using a validated tool were conducted for patients who were arrived DOA. As a comparison, cases during the same months prior to the COVID-19 were analyzed for respective waves. Events were counted, and proportions and frequencies are reported for each time period.

**Results:**

A total of 4,480 ACS cases were reviewed; 1,216 cases during March-July 2019, 804 cases in the same months of 2020 (33.8% decrease); 1,304 cases in August 2019-January 2020 and 1,157 in the corresponding months of 2020 and 2021 (11.2% decrease). There was no observed change in the baseline characteristics of patients with ACS or their symptom-to-door time, and in-hospital mortality was unchanged across all time periods. There were 218 DOA cases in pre-pandemic months and 360 cases during the pandemic. The pre-pandemic rate of DOA was 12/1000 emergency patients (95% CI 10–13) compared to 22/1000 (95% CI 22–27) during the pandemic (30/1000in the 1^st^ wave and 17/1000 during 2^nd^ wave). On VA, CVD was found to be the major cause of death during both time periods.

**Conclusion:**

At a cardiac hospital in Pakistan, the COVID-19 pandemic was associated with a reduction in ACS hospitalization and an increased DOA rate.

## Introduction

In 2020, the sudden surge of patients suffering from COVID-19 disrupted normal patterns of global health care delivery. With the increase in the number of COVID-19 cases admitted to hospitals, a decline in the hospital census of acute clinical illnesses such as appendicitis, stroke, and myocardial infarction (MI) was observed in multiple countries [1–3]. The fear of nosocomial spread is believed to have led to hospital avoidance, even for acute conditions [4, 5]. A 40% drop in emergency room visits in the United Kingdom was documented during the first peak of the pandemic in 2020 [4]. A perceived reduction of 40–60% in ST elevated myocardial infarction (STEMI) cases was reported by nurses and physicians in a global survey, with an increase of 40–60% in late presentation of STEMI [6].

This change in health care seeking resulted in a substantial increase in out-of-hospital deaths due to cardiac arrest: in the United States (US) during the pandemic, the incidence of out-of-hospital cardiac arrest (OHCA) doubled from 20 to 47 cases per 100,000 [7]. Similar findings were reported in France, where the incidence of OHCA was twice as high during the pandemic period compared to pre-pandemic similar months of the year [7].

COVID-19 preventive measures such as mandated "lockdown" with resulting transport disruption have been reported to decrease access to care for human immune deficiency virus (HIV) patients in low and middle-income countries (LMICs) [8]. The indirect effect of COVID-19 on cardiovascular morbidity and mortality could be more pronounced in Pakistan, where patients have limited healthcare literacy and limited access to emergency medical services (EMS) for acute life-threatening conditions such as MI. There is no EMS system in Pakistan. Ambulances services are limited; ambulances are not equipped with essential medical devices, and ambulance staff is not trained to provide emergency treatment. Hence, the patients did not receive any resuscitation during the transfer from home to hospital. As a result, the first point of medical intervention may be the health facility, and patients commonly arrive having died during transport.

In Pakistan, the first "wave" of COVID-19 spread was recorded between March and July 2020, and the second (2^nd^) was between August 2020 and January 2021[9]. The worst affected city was Karachi, which recorded the highest number of cases in the country. However, there are no national or regional mortality or emergency services registries in Karachi or Pakistan[10], and no cause-specific data for ACS or OHCA have yet been published.

To explore these questions, we conducted a study to compare the frequency of ACS hospitalization, ACS severity, and dead on arrival (DOA) due to presumed OHCA in the emergency room (ER) at the Tabba Heart Institute during pre and intra-pandemic periods and investigated possible causes of death employing verbal autopsy (VA).

## Methodology

Tabba Heart Institute (THI) is a 160 bed, single-specialty cardiac tertiary center in Karachi, Pakistan. THI ER cares for approximately 1600 patients per month and serves as one of the major referral centers in Karachi for STEMI percutaneous coronary interventions (PCI). In 2020, the hospital employed 15 general cardiologists and seven interventional cardiologists and performed an average of 1,600 PCI procedures annually. The hospital maintains an electronic medical record (EMR) system for all patients receiving emergency, outpatient, and inpatient services.

A comparative study was undertaken of patients who were dead on arrival (DOA) or who were admitted with the acute coronary syndrome (ACS) from the ER of THI. We compared the 1^st^ COVID-19 wave with its corresponding pre-pandemic months in 2019 and the 2^nd^ wave with a mirrored pre-pandemic time period of identical length in an attempt to more effectively compare case counts. The pre-pandemic periods evaluated cases from March to July 2019 as well as August 2019 to January 2020, the 1^st^ wave of the pandemic from March to July 2020, and the 2^nd^ wave from August 2020-January 2021.

Data for this study was extracted from the Chest Pain-MI Registry™ and CathPCI Registry^®^ maintained at THI, which is affiliated and benchmarked by the National Cardiovascular Disease Registry (NCDR^®^), USA. Another source of data utilized was patients’ electronic medical records (EMRs), which were mainly used for the patients who were DOA. The hospital protocol is not to attempt resuscitation for patients reported to be unresponsive for >45 minutes before arrival and for patients displaying rigor mortis. We defined DOA as patients with no recordable blood pressure and pulse and documented as DOA by an emergency physician.

Demographic data were collected for all patients, as well as the history of risk factors for coronary disease (e.g., hypertension, diabetes, dyslipidemia), known diagnosis of coronary artery disease, previous cardiac events, and the severity of disease at presentation (e.g., cardiogenic shock). For ACS patients, symptom-to-door time, post-PCI complications, and in-hospital mortality were also collected.

For DOA, death certificates and doctors’ notes were reviewed by the research team. The family of the deceased was approached via contact information documented in the EMR. Trained medical officers conducted a VA after verbal consent for participation was obtained. The Population Health Metrics Research Consortium’s (PHMRC) shortened verbal autopsy questionnaire for adults was modified and used for the telephonic interviews [11]. The questionnaire was further shortened out of concern that discussion might lead to emotional distress to the family. See S1 Appendix. The study was reviewed by the Institutional Review Board of Tabba Heart Institute and was approved for verbal consent and ethical clearance (OHRP Reg#IORG0007863).

VA is not routinely performed at THI and was expressly carried out for the purpose of this investigation for both pre and intra-pandemic deaths. Information collected on VA was combined with risk factors documented in the EMR, and a likely cause of death was assigned as described below. The algorithm to assign the cause of death was developed by consensus of SS (epidemiologist), SA (cardiologist and epidemiologist), and AP (intervention cardiologist). The cause of death was classified into the following categories: possible CVD, likely CVD, COVID/respiratory tract infection (RTI), others, and unclassified. Before death, any patient who reported chest pain (with or without dyspnea) was classified as "likely" CVD. Patients with dyspnea alone preceding death and past history or risk factors for CVD were classified as "possible" CVD death. All patients with fever—with or without other symptoms—were categorized as COVID/RTI. Patients lacking specific reported signs and symptoms immediately before death but who had a past history of cerebrovascular disease, renal disease, cancer, or other non-cardiovascular diseases such as chronic obstructive pulmonary disease were classified as "other." Cases lacking reported signs or symptoms preceding death and without prior recorded clinical data, as well as those for whom a VA could not be conducted, were left unclassified.

Statistical analysis: Frequencies and percentages of the categorical outcomes were reported and proportion difference with 95% confidence interval (CI) between the 1^st^ wave and matched months of pre-COVID times, and 2^nd^ wave and its corresponding time-periods was estimated. Median and intra-quartile range (IQR) of time from onset of symptoms to hospital arrival was calculated between the time of pre-pandemic and intra-pandemic duration. Incidence proportion (IP) was calculated by taking ACS or DOA as numerator and ER volume of the same duration as the denominator. The incidence ratio/risk ratio for pandemic (IR/RR) was calculated by taking IP during the pandemic and dividing it by IP during pre-pandemic. The cause-specific mortality rates for CVD during pandemic and pre-pandemic were also calculated (death due to CVD/total number of patients attending ER). All rates are reported with 95% CIs.

## Results

There were 18,209 patient visits to ER during pre-pandemic months and 15,790 during pandemic months (13.2% reduction). The final ACS data set consisted of 4,480 cases; 1,216 cases during March-July 2019, 804 cases in the same months of 2020, 1304 cases in August 2019-January 2020, and 1157 in the corresponding months of 2020 and 2021. There was no difference in the presentation of cases between the 1^st^ wave and pre-COVID months and the 2^nd^ wave and its pre-COVID time with respect to age, gender, or risk factors of ACS **(Table 1)**.

**Table 1:** Baseline characteristics of ACS patients between pre-pandemic months matched to 1^st^ and 2^nd^ COVID waves.

Variables	1^st^ wave’s corresponding pre-pandemic months n = 1216 n (%) Mean±SD	1^st^ wave n = 803 n (%) Mean±SD	p-value	2^nd^ wave’s corresponding pre-pandemic months n = 1304 n (%) Mean±SD	2^nd^ wave n = 1157 n (%) Mean±SD	p-value
Age (years)	59.1±12.0	59.0±11.3	0.86	60.3±11.5	61.1±11.6	0.08
Men	879 (72.3)	593 (73.8)	0.44	971 (74.5)	851 (73.6)	0.60
Dyslipidemia	500 (41.1)	323 (40.2)	0.68	518 (39.7)	474(41.0)	0.53
Hypertension	838 (68.9)	515 (64.1)	0.02	868 (66.6)	762 (65.9)	0.71
Diabetes mellitus	601 (49.4)	407(50.7)	0.57	657 (50.4)	622 (53.8)	0.09
Tobacco use	302 (24.7)	205 (25.5)	0.72	326 (24.9)	304 (26.2)	0.47

Waves were compared to their corresponding months of the pre-COVID era; chi-square test was applied for categorical variables and student’s t-test for continuous variables; significant p-value <0.05; NS (non-significant) = p-value >0.05.

**Fig 1:**
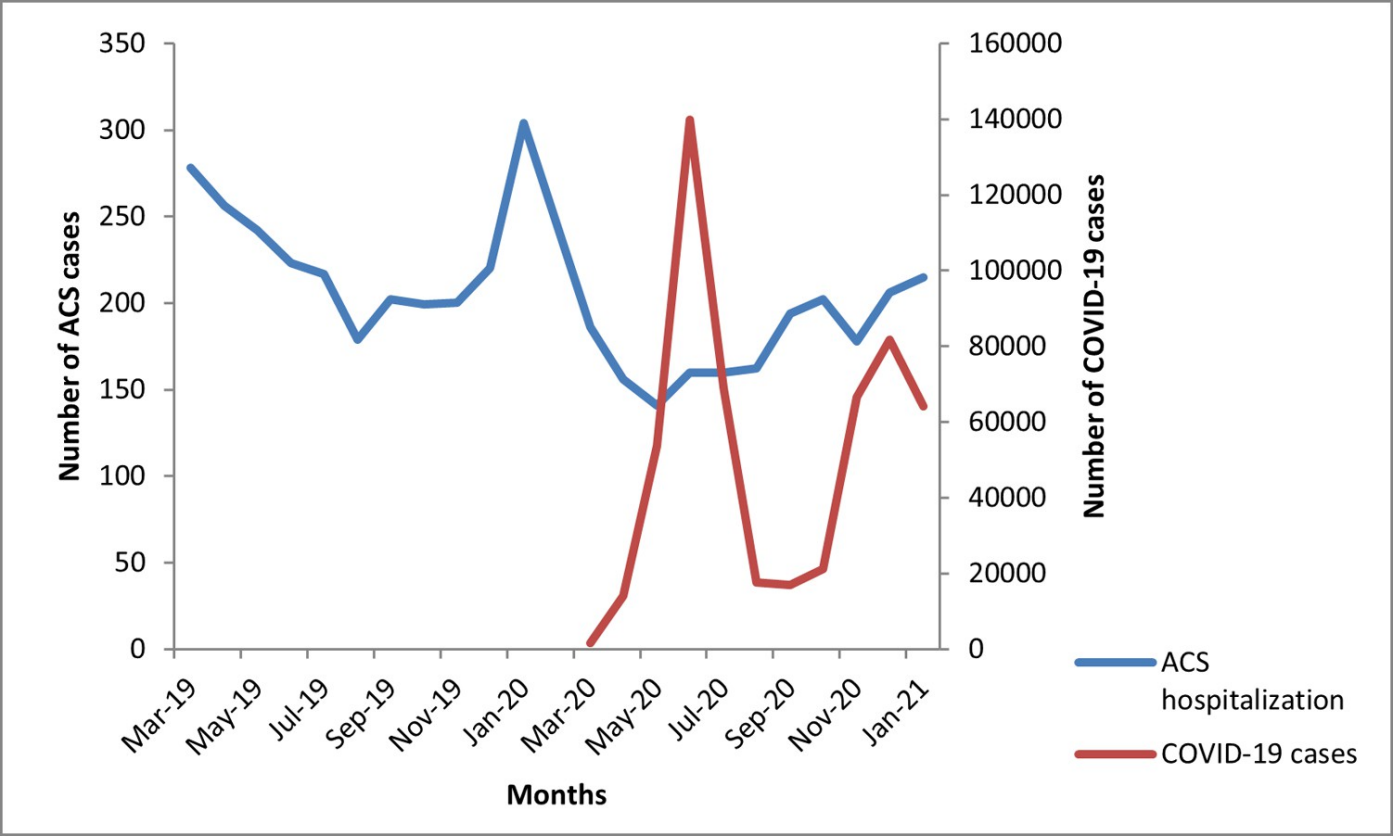
ACS cases between pre-pandemic vs. pandemic months and number of COVID-19 cases in Pakistan.

There was an overall reduction of 22.1% in ACS hospitalization in the pandemic period; 33.9% cases during 1^st^ wave of pandemic compared to the corresponding months of pre-pandemic era and 11.2% decrease during 2^nd^ wave and its matched months before pandemic **(Fig 1)**. The reduction in the number of ACS cases was following the pattern of COVID-19 cases in Pakistan. With the sharp increase in the number of COVID-19 cases during 1^st^ wave, ACS cases declined. However, during the second wave of COVID-19, the decline in ACS cases presenting to the hospital was less pronounced than during the 1^st^ wave, but the lowest number of cases was observed in association with the peak of COVID cases during the 2^nd^ wave (Fig 1).

**Fig 2:**
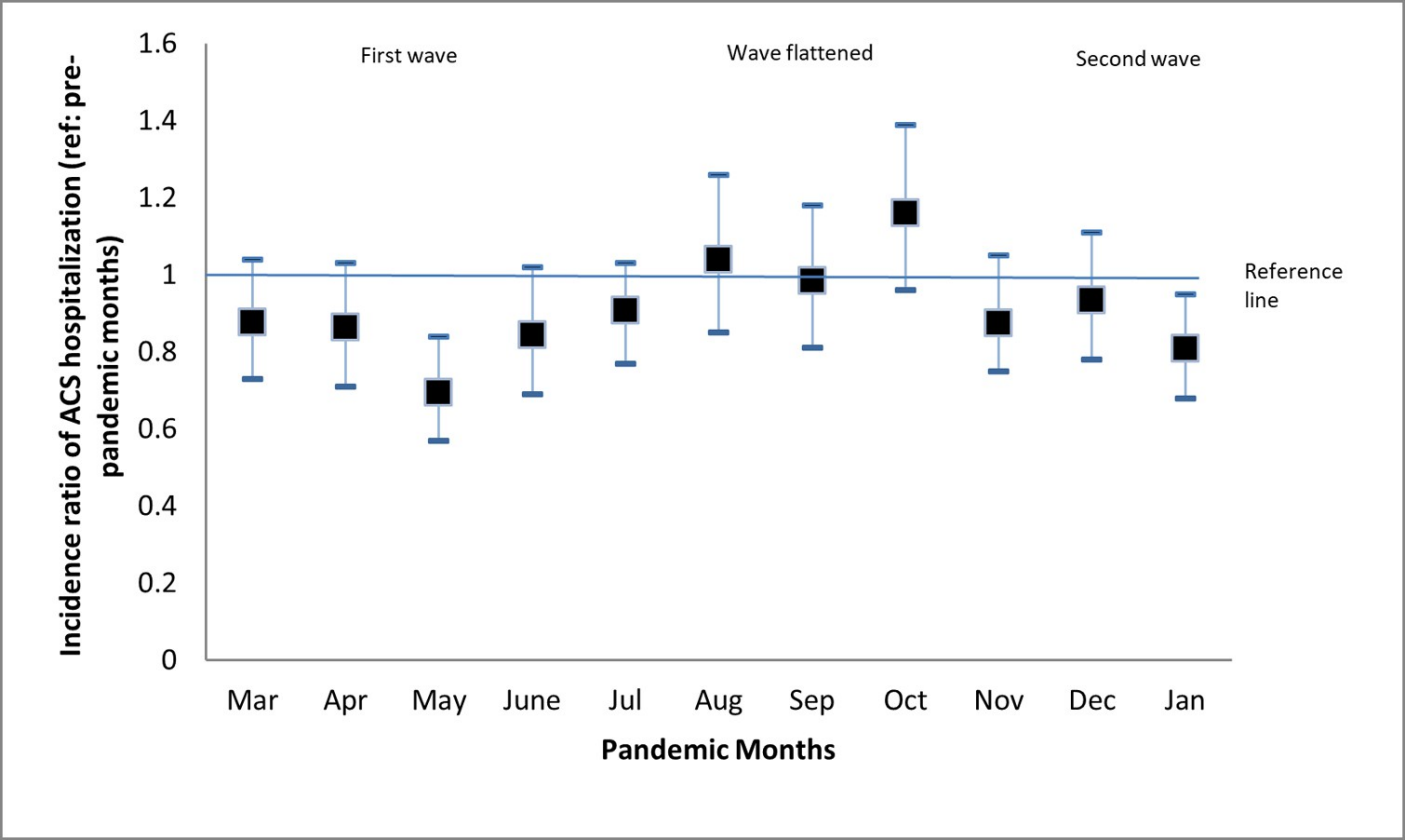
Incidence ratios of ACS hospitalization between pre-pandemic vs. pandemic months.

The IP of ACS during pre-pandemic was 140/1000 patients presenting to the ER compared to 120/1000 during the pandemic (IR 0.90 [95% CI 0.84–0.94]). The incidence ratio during the 1^st^ COVID wave was 0.83 (95% CI 0.76–0.9), and during the 2^nd^ COVID wave, it was 0.95 (95% CI 0.88–1.02) **(Fig 2)**.

Although all categories of ACS presentation were reduced during the 1st COVID wave, there was a significant and more marked reduction in the lower risk categories. The low-risk chest pain patient presentation was reduced by 72%, unstable angina 25%, and NSTEMI reduced by 20% during the 1^st^ wave compared to the similar months during the pre-pandemic era. Such changes were not as pronounced in the 2^nd^ wave when compared to pre-pandemic months **(Table 2)**. The incidence of STEMI hospitalization remained unchanged between the COVID-19 waves and their respective pre-pandemic months **(Table 2)**.

**Table 2:** Types of ACS patients admitted during 1^st^ and 2^nd^ COVID waves and matched pre-pandemic months.

Types of cases	1^st^ wave’s corresponding pre-pandemic time period N = 1216 N (%) Median (IQR)	1^st^ wave N = 803 N (%) Median (IQR)	IR (95% CI)	p-value	2^nd^ wave’s corresponding pre-pandemic time period N = 1304 N (%) Median (IQR)	2^nd^ wave N = 1157 N (%) Median (IQR)	IR (95% CI)	p-value
Low-risk chest pain	83 (6.8)	19 (2.4)	0.28 (0.17, 0.47))	<0.01	63 (4.8)	47 (4.1)	0.83 (0.54,1.1)	0.35
Unstable angina	138 (11.3)	82 (10.2)	0.75 (0.57, 0.98)	0.04	83 (6.4)	115 (9.9)	1.5 (1.1, 1.9)	0.03
NSTEMI	645 (53.0)	414 (51.6)	0.80 (0.72, 0.91)	0.03	733 (56.2)	610 (52.8)	0.89 (0.80, 0.99)	0.05
STEMI	350 (28.8)	288 (35.9)	1.0 (0.89, 1.2)	0.10	425 (32.6)	384 (33.2)	0.95 (0.84, 1.1)	0.11

-Waves were compared to their corresponding months of pre-COVID era; chi-square test was applied; significant p-value <0.05; NS (Non-significant) = p-value > 0.05.

-Low-risk chest pain is hemodynamically stable patient, with no significant sign and symptom of MI, and no ECG or biomarker evidence of MI.

There was an increase in the proportion of patients (1.9%) who presented with cardiac arrest during the 1^st^ wave compared to pre-pandemic months. In addition, there was a 6.1% increase in catheterization and percutaneous coronary intervention procedures during 1^st^ wave compared to its corresponding pre-pandemic months **(Table 3)**. We did not observe a difference in symptom-to-door time across 1^st^ wave and its pre-pandemic months and 2^nd^ wave and its corresponding pre-pandemic time period **(Table 3)**. Overall, patient presentation during 2^nd^ wave was not different from its corresponding months of the pre-pandemic era. There was also no difference in in-hospital mortality between pandemic and pre-pandemic months during both waves **(Table 3)**.

**Table 3:** Clinical presentation, management, and hospital outcome of ACS patients admitted during 1^st^ and 2^nd^ COVID waves and matched pre-pandemic months.

Variables	1^st^ wave’s corresponding pre-pandemic time period N = 1216 N (%) Median (IQR)	1^st^ wave N = 803 N (%) Median (IQR)	% difference (95% CI)	p-value	2^nd^ wave’s corresponding pre-pandemic time period N = 1304 N (%) Median (IQR)	2^nd^ wave N = 1157 N (%) Median (IQR)	% difference (95% CI)	p-value
Heart failure	185 (15.2)	104 (13.0)	-2.2 (-5.2, 0.8)	0.16	238 (18.3)	202 (17.5)	-0.8 (-3.8, 2.2)	0.60
Cardiac arrest	18 (1.5)	27 (3.4)	1.9 (0.4, 3.3)	<0.01	27 (2.4)	16 (1.5)	-0.9 (-1.9, 0.1)	0.10
Cardiogenic shock	22 (1.8)	23 (2.9)	1.1 (-0.2,2.4)	0.10	37 (2.8)	25 (2.2)	-0.6 (-1.8, 0.6)	0.34
Cath-lab utilization:				0.01				<0.01
No visit	207 (17.0)	106 (13.2)	-3.8 (-7.6, -1.3)		172 (13.2)	213 (18.4)	5.2 (2.3, 8.0)	
Diagnostic cath	293 (24.1)	196 (24.4)	0.3 (-3.5, 4.1)		335 (25.6)	259 (22.3)	-3.3(-6.6, 0.07)	
Revascularization:	716 (58.8)	501 (62.3)	3.5 (-0.8, 7.8)		797 (61.1)	685 (59.2)	-1.9(-5.7, 1.9)	
Both cath and PCI	544 (44.7)	408 (50.8)	6.1(1.6, 10.5)		608 (46.6)	534 (46.1)	-0.5(-4.4, 3.4)	
CABG	172 (14.2)	93 (11.6)	-2.6(-5.5, 0.3)		189 (14.6)	151 (13.2)	-1.4(-4.1, 1.3)	
Symptom to door time (minutes)*	164.5 (91, 356.2)	192.0 (98.5, 456.7)	-	0.79	200.0 (107.0, 445.5)	194.0 (118.0, 470.2)	-	0.37
In-hospital mortality	48 (3.9)	32 (4.0)	0.1(-1.6, 1.8)	0.91	54 (4.1)	49 (4.2)	0.1 (-1.4, 1.6)	0.90

*data for n = 16 is missing for Killip class; medians were compared using Mann Whitney U-test, and p values are reported; chi-square was applied for categorical variables; p-value <0.05 is significant; NS (non-significant) = >0.05.

### DOA results

**Fig 3:**
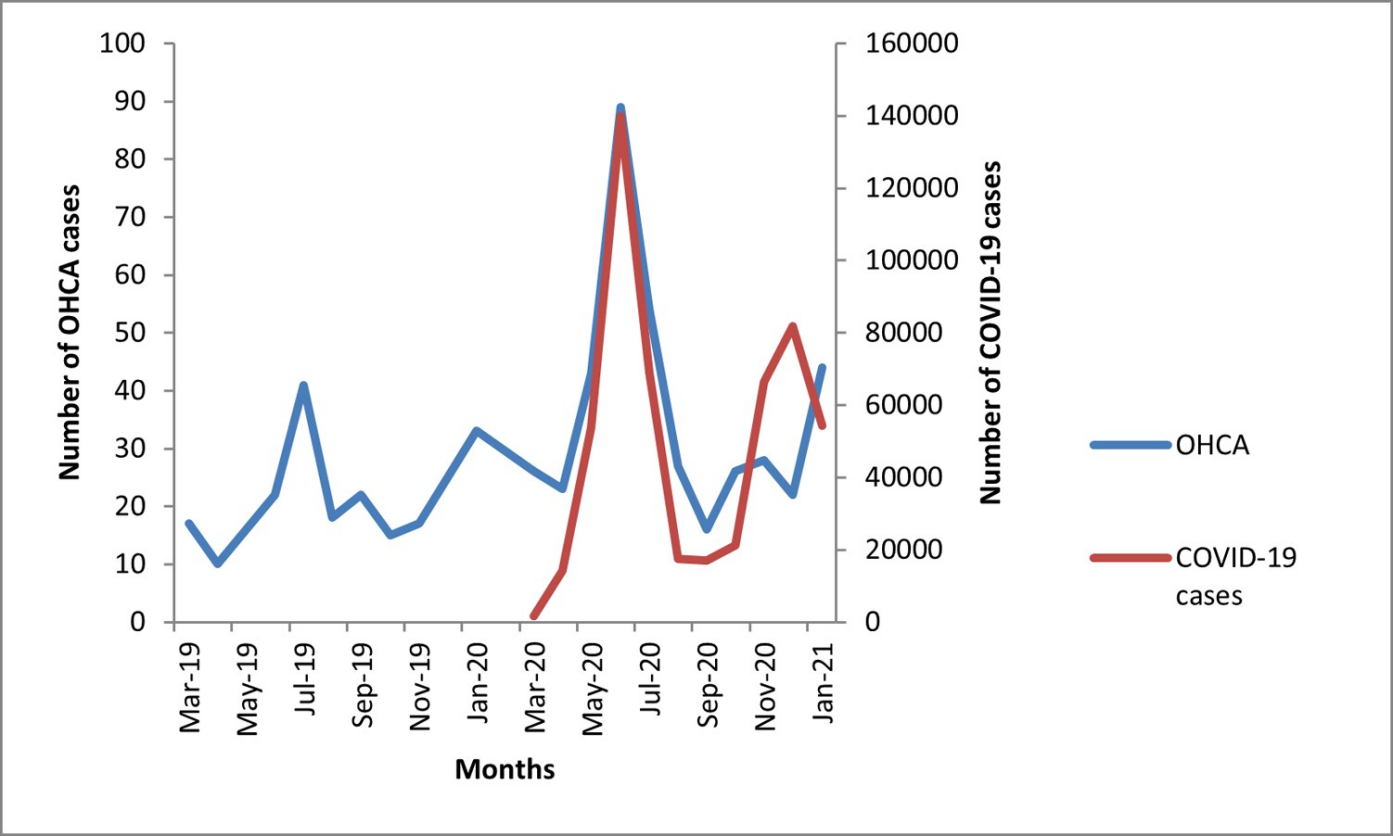
COVID-19 cases in Pakistan, and DOA at study-hospital between pre-pandemic vs. pandemic months.

There were 218 DOA cases in pre-pandemic months out of 18,209 patients attending ER and 360 DOA cases during the pandemic with 15,970 ER visits. Thus, the IP of DOA cases during the pre-pandemic era was 12/1000 patients attending ER (IP 95% CI 10, 13 cases), which increased to 22/1000 (IP 95% CI 19, 24 cases) during pandemic (RR 1.8, 95% CI 1.5, 2.1) **(Fig 3)**.

The IP of DOA in 1^st^ wave was 30/1000 (204/6630) and 17/1000 (156/9160) during 2^nd^ wave. Among the DOA cases, the mean age of DOA during the pandemic was 2.7±1.9 years younger (p-value < 0.05). There was no statistical difference across the two time periods in terms of gender, co-morbidities, tobacco use, and history of coronary revascularization procedures except hypertension and mean age **(Table 4)**.

**Table 4:** Baseline characteristic of DOA during the pandemic and pre-pandemic era.

Variables	Pandemic era	Pre-pandemic era	p-value
	N = 360	N = 218	
	N (%)	N (%)	
	Mean±SD	Mean±SD	
Age (years)	61.7±14.4	64.4±12.5	0.02
Men	227 (63.1)	141 (64.7)	0.69
Prior PCI	24 (11.5)	28 (14.0)	0.46
Prior CABG	13 (6.2)	18 (9.0)	0.28
Dyslipidemia	55 (26.2)	35 (17.9)	0.06
Hypertension	111 (51.6)	127(63.5)	0.02
Diabetes mellitus	158 (48.8)	103 (50.2)	0.84
History of congestive heart failure	25 (15.0.1)	32 (16.3)	0.74
Tobacco use	19(8.4)	19 (9.6)	0.38

*Data of 157 patients missing; chi-square test for categorical and student’s t-test for continuous variables is used;p-value <0.05 is significant; NS (non-significant) = p-value >0.05.

We completed the verbal autopsy of 209 (95.8%) cases from pre-pandemic months and 339 cases (94.1%) from pandemic months.

**Fig 4:**
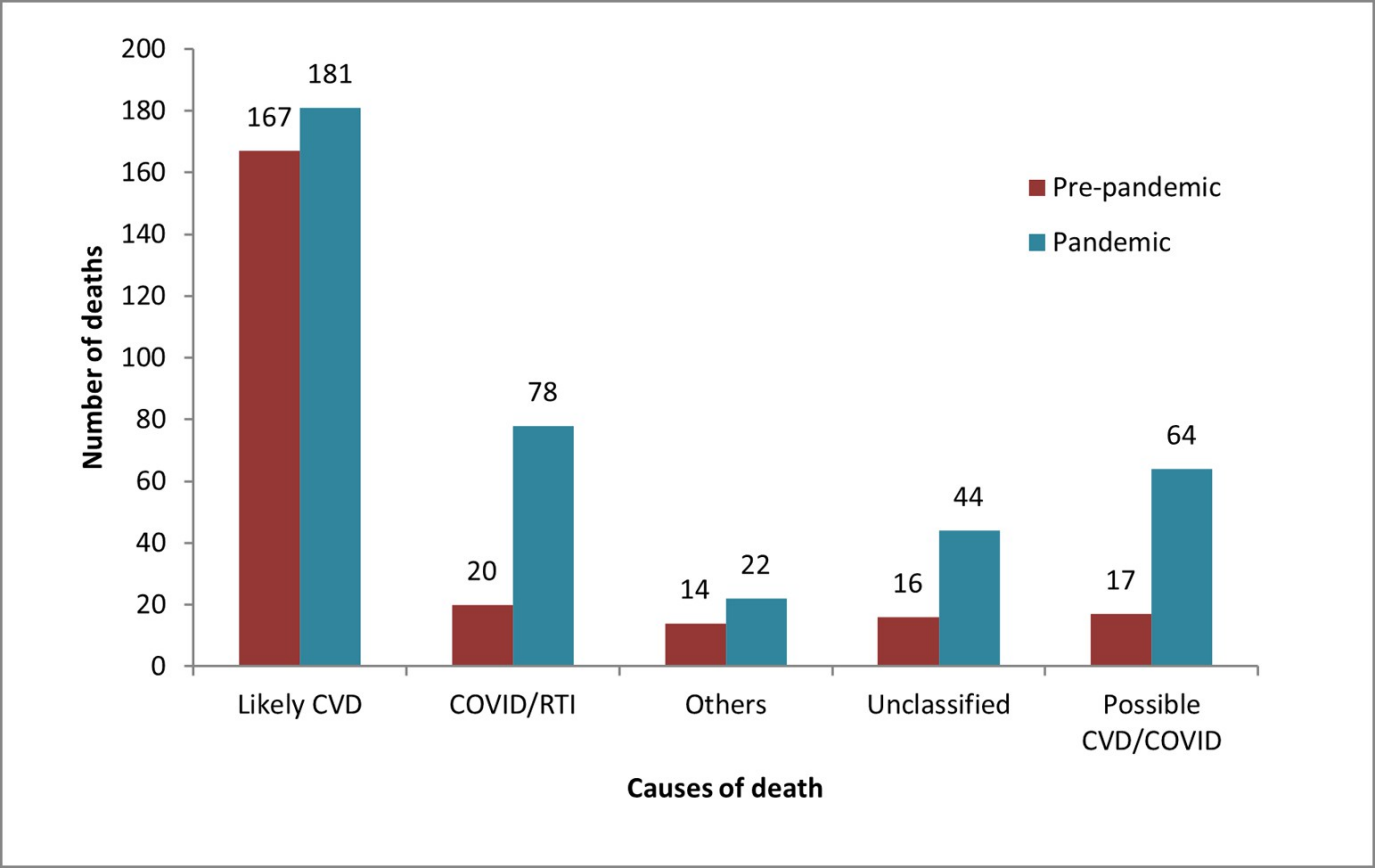
Comparison of causes of DOA between pre-pandemic vs. pandemic months.

CVD was the major cause of death during both time periods. The cause-specific mortality rate of CVD among DOA patients increased from 9/1000 patients (95% CI [8, 11]) in the pre-pandemic period to 10/1000 (95% CI [9, 12]) during the pandemic. There were 21% (75/360)deaths due to COVID-19 infection, making it the second-highest cause of death among DOA cases during the pandemic. There were 16.1% (58/360) of the deaths during the pandemic that were possible CVD/COVID. As we could not conduct VA of all deaths, there were 11.6% deaths during the pandemic and 6.4% during pre-pandemic that cannot be classified in any category **(Fig 4)**.

## Discussion

We observed a 13% decrease in ER visits and a 22% decrease in the number of ACS admissions during the 2020 COVID-19 pandemic compared to a matched period during the preceding year. A greater reduction in ACS was observed during the 1^st^ wave of COVID-19 in the region as compared to the 2^nd^ wave (33.9% reduction vs. 11.2%). There was an increase in the proportion of cardiac arrests during the 1^st^ wave of pandemic. The probability of DOA presentation during the pandemic was approximately doubled when compared to matched pre-pandemic periods and an increase in DOA due to CVD (11/1000 vs.9/1000 patients) was observed on verbal autopsy.

The reduction in ACS admissions during the COVID pandemic observed in our study is consistent with the recently published literature. Declines in ACS hospitalization have been reported by multiple countries irrespective of their COVID infection rates. Reports from the US, the country with the highest number of COVID-19 cases in 2020, showed a 70% reduction in ACS admissions, while New Zealand, which maintained very low infection rates, observed a 28% reduction in ACS admissions [12, 13]. A study from Swiss Ticino Canton showed an increase in the symptom-to-door time due to delay in call for EMS services despite the low COVID infection rate in the region [14]. The decrease in ACS hospitalization has been variously attributed to overwhelmed health systems due to COVID patients, fear of nosocomial COVID-19 exposure among patients, and societal restrictions related to lockdown. There may also be a decrease in ACS incidence due to reduced pollution, stress, and other lifestyle changes [3, 15–17].

Similar to our findings, national-level data from Denmark also reported a reduction in presentation of low-risk ACS patients during pandemic compared to pre-pandemic years [15]. Chan et al. did not find any change in STEMI presentation to a hospital in New Zealand despite a 28% reduction in ACS admissions, consistent with our results [12]. A pooled analysis of 38 studies from multiple countries on ACS hospitalization found a significant reduction in STEMI (Incidence rate ratio (IRR) 0.74 95% CI [0.73–0.75]) hospitalization similar to an overall reduction of ACS cases (IRR 0.73 95% CI [0.72–0.73]) [18]. The absence of change in STEMI hospitalization in our results may reflect patient avoidance of health facilities for less sick patients, with preserved utilization for critical disease severity. There appears to be an increase in cardiac catheterization (CAG) and percutaneous coronary intervention (PCI) procedures among ACS patients at THI. The finding is not aligned with the literature, where a reduction in STEMI cases was highlighted, resulting in decreased cath lab activation during the pandemic [19]. It might also be explained by decreased overall hospital volumes during the pandemic due to a reduction in ACS and other CVD patients, coupled with the availability of PPE, making it more possible to utilize the cath lab for STEMI patients. Additionally, a decrease in volumes of low-risk ACS patients could have led to an increased probability of invasive evaluation and PCI for higher acuity patients.

In our study, the relative risk of DOA increased during the pandemic period (RR 1.9, 95% CI [1.6, 2.2]), which supplements the literature on OHCA from multiple countries [3, 7, 20]. Literature published from higher-income countries compared the rate of survival of patients who received CPR from EMS during the pandemic and pre-pandemic months, and a higher rate of failed to resuscitate and DOA was observed during the 2020 pandemic [20, 21]. In the US, a decrease in ACS cases was coupled with a 59% increase in OHCA [22]. The increase in OHCA may be due to fewer patients with ACS presenting to the hospitals [3]. COVID-19 may directly be contributing to this. It is well established that the pro-thrombotic state is associated with COVID-19 with a resultant increase in OHCA related to ACS or pulmonary embolism [22].

Lockdown was the major preventive measure implemented by almost all the countries to limit the spread of COVID-19 infection [23]. It is postulated that lockdown itself with reduced mobility and access to healthcare has been detrimental to patients with health emergencies such as ACS and contributed to the reduction in hospitalization and increase in OHCA [17]. This is supported by our results of greater difference between ACS and DOA rates during 1^st^ wave and pre-pandemic time compared to 2^nd^ wave and its identical pre-pandemic times in our study. Ambulance networks are limited in Pakistan, and public or private vehicles are the main source of hospital transportation. During the 1st wave, public transport services were closed by government mandate, making it difficult for patients to travel to health care facilities. General perception of COVID-19 hazards also changed between the two waves, as patients may have become less concerned about their personal risk of contracting the disease. Physical distancing, use of face masks, and stay at home instructions were less stringently followed in later periods.

The major contributor of DOA in our study was CVD, followed by presumed COVID-19 infection. As the study site is a cardiac-only health facility, CVD was the leading cause of death during all study periods. There has been an increase in the CVD-specific mortality rate during the COVID-19 pandemic, consistent with other researchers’ conclusions [20]. Baldi and Marijon reported in their respective studies that direct effects of COVID-19 infection on the cardiovascular system, indirect effects of barriers to health facility access, and patient anxiety and stress may have contributed to an increase in deaths due to CVD during the pandemic [3, 20]. These studies also acknowledged that, at times, it is difficult to identify the definitive cause of death prior to arrival due to similar symptoms (dyspnea) and entwined pathophysiological pathway of COVID-19 and MI or arrhythmias [3, 20].

We believe that our study has several important strengths that bear consideration: data was extracted from a standardized registry, and two waves of the pandemic were compared with the matched pre-pandemic months. The time period of both pandemic and pre-pandemic covered almost a year (March-January) and, therefore, should mitigate seasonal variation in ACS cases [24]. Hence, we believe that the changes in the rate of ACS and DOA are reflective of the impact of COVID-19. An important limitation of our study is potential recall bias in verbal autopsy data when comparing cases from 2020–2021 and 2019. Also, we did not have VA data for 10% of the cases from pre-pandemic and 12% from intra-pandemic months. As mentioned earlier, verbal autopsy is not a routine practice at THI. In the absence of national or regional autopsy data or standardized mortality registers, we believe that using a short standardized verbal autopsy instrument was the best possible method to determine the likely cause of death. For efficiency, we further shortened the standardized VA short tool (See S1 Appendix) and therefore advise readers to interpret our VA results cautiously. Another limitation is data regarding cardiac arrest are not collected according to Utstein style.

## Conclusion

We observed a reduction in ACS hospitalization and an increase in DOA presentation during the COVID-19 pandemic at our cardiac specialty hospital. The reduction in ACS cases and increase in DOA presentation were more marked during the 1^st^ wave than during the 2^nd^ wave of COVID-19 spread. In the context of care in an LMIC, further investigations are required to assess the health risk to patients with CVD to understand and mitigate the impacts of pandemic spread on emergency services and access to health care facilities.

## Supporting information

S1 Appendix Acute coronary syndrome cases during COVID-19 pandemic compared to pre-pandemic era at an urban tertiary care hospital in Pakistan. (DOCX)

## Abbreviations

ACS: Acute coronary syndrome
CVD: Cardiovascular disease
CA: Cardiac arrest
CI: Confidence interval
DOA: Dead on arrival
EMRs: Electronic medical records
EMS: Emergency medical services
HIV: Human immune deficiency virus
IP: Incidence proportion
IQR: Intra-quartile range
IR: Incidence ratio
IRR: Incidence rate ratio
MI: Myocardial infarction
NCDR®: National Cardiovascular Disease Registry
DOA: Out of hospital cardiac arrest
PCI: Percutaneous coronary intervention
PHMRC: Population Health Metrics Research Consortium’s
RR: Risk ratio
STEMI: ST elevated myocardial infarction
THI: Tabba Heart Institute
US: United States
VA: Verbal autopsies
