# Supplementary material for: Cases of acute coronary syndrome and presumed cardiac death prior to arrival at an urban tertiary care hospital in Pakistan during the COVID-19 pandemic
Source: PLoS One. 2022 Feb 3;17(2):e0263607. doi: 10.1371/journal.pone.0263607 (PMC8812872; doi:10.1371/journal.pone.0263607)
Supplement: S1 Appendix — (DOCX) [file pone.0263607.s001.docx]

S1 Appendix

**Acute coronary syndrome cases during COVID-19 pandemic compared to pre-pandemic era at an urban tertiary care hospital in Pakistan**

**SECTION 1: HISTORY OF CHRONIC CONDITIONS OF THE DECEASED**

| 1.1 | Was ______ ever told by a health professional that he or she ever suffered from one of the following?  ـــــــــــــــــــکو کبھی کیسی ڈاکٹر نےبتایا ان کو نیچے دی گئی بیماریوں میں سے کوئی بیماری لاحق ھے؟کیا | | |
| --- | --- | --- | --- |
| 1.2 | Diabetes  ذیابطیس | 1. Yes   1۔ھاں   1. No   2-ناں  8. Refused to answer  8۔ جواب دینے سے انکار کر دیا  9. Don’t know  9۔ نھیں پتہ | ****  ****  ****  **** |
| 1.3 | Heart Disease  دل کی بیماری | 1. Yes   1۔ھاں   1. No   2-ناں  8. Refused to answer  8۔ جواب دینے سے انکار کر دیا  9. Don’t know  9۔ نھیں پتہ | ****  ****  ****  **** |
| 1.4 | Stroke  فالج | 1. Yes   1۔ھاں   1. No   2-ناں  8. Refused to answer  8۔ جواب دینے سے انکار کر دیا  9. Don’t know  9۔ نھیں پتہ | ****  ****  ****  **** |
| 1.5 | Asthma  دمہ | 1. Yes   1۔ھاں   1. No   2-ناں  8. Refused to answer  8۔ جواب دینے سے انکار کر دیا  9. Don’t know  9۔ نھیں پتہ | ****  ****  ****  **** |
| 1.6 | Cancer  کینسر | 1. Yes   1۔ھاں   1. No   2-ناں  8. Refused to answer  8۔ جواب دینے سے انکار کر دیا  9. Don’t know  9۔ نھیں پتہ | ****  ****  ****  **** |
| 1.7 | COPD (Chronic Obstructive Pulmonary Disease)  سانس کی پرانی بیماری | 1. Yes   1۔ھاں   1. No   2-ناں  8. Refused to answer  8۔ جواب دینے سے انکار کر دیا  9. Don’t know  9۔ نھیں پتہ | ****  ****  ****  **** |
| 1.8 | Epilepsy  مرگی | 1. Yes   1۔ھاں   1. No   2-ناں  8. Refused to answer  8۔ جواب دینے سے انکار کر دیا  9. Don’t know  9۔ نھیں پتہ | ****  ****  ****  **** |
| 1.9 | Tuberculosis  ٹی بی | 1. Yes   1۔ھاں   1. No   2-ناں  8. Refused to answer  8۔ جواب دینے سے انکار کر دیا  9. Don’t know  9۔ نھیں پتہ | ****  ****  ****  **** |

**SECTION 2: SYMPTOM CHECKLIST**

| 2.1 | Did _____ have a fever?  کیا ــــــــــــــــــ کو بخار تھا؟ | 1. Yes   1۔ھاں   1. No   2-ناں  8. Refused to answer  8۔ جواب دینے سے انکار کر دیا  9. Don’t know  9۔ نھیں پتہ | ****  ****  ****  **** |
| --- | --- | --- | --- |
|  | *If "No" or "Don’t know” or “Refused to answer” go to 2.4*  *اگر 'ناں' یا '* نھیں پتہ' تو 4۔2 پہ جائیں | | |
| 2.2 | How severe was the fever?  بخار کتنا شدید تھا؟ | 1. Mild   1۔ ہلکا   1. Moderate   2۔ درمیانہ   1. Severe   3۔ شدید  88. Refused to answer  8۔ جواب دینے سے انکار کر دیا  9. Don’t know  9۔ نھیں پتہ | ****  ****  ****  ****  **** |
| 2.3 | What was the pattern of the fever?  بخار کس دورانیہ کا تھا؟ | 1. Continuous   1۔ مستقل   1. On and off   2۔ اترتا چڑھتا تھا   1. Only at night   3۔ صرف رات کو چڑھتا تھا  8. Refused to answer  8۔ جواب دینے سے انکار کر دیا  9. Don’t know  9۔ نھیں پتہ | ****  ****  ****  ****  **** |
| 2.4 | Did _____ have a cough?  کیا ــــــــــ کو کھانسی تھی؟ | 1. Yes   1۔ھاں   1. No   2-ناں  8. Refused to answer  8۔ جواب دینے سے انکار کر دیا  9. Don’t know  9۔ نھیں پتہ | ****  ****  ****  **** |
|  | *If “No” or “Don’t know” or “Refused to answer” go to 2.8*  *اگر 'ناں' یا '* نھیں پتہ' تو7۔2 پہ جائیں | |  |
| 2.5 | Did the cough produce sputum?  کیا کھانسی میں بلغم تھا؟ | 1. Yes   1۔ھاں   1. No   2-ناں  8. Refused to answer  8۔ جواب دینے سے انکار کر دیا  9. Don’t know  9۔ نھیں پتہ | ****  ****  ****  **** |
| 2.6 | Did _____ cough blood?  کیا کھانسی میں خون تھا؟ | 1. Yes   1۔ھاں   1. No   2-ناں  8. Refused to answer  8۔ جواب دینے سے انکار کر دیا  9. Don’t know  9۔ نھیں پتہ | ****  ****  ****  **** |
| 2.7 | Did _____ have difficulty breathing?  کیا ــــــــــــ کو سانس لینے میں دشواری تھی؟ | 1. Yes   1۔ھاں   1. No   2-ناں  8. Refused to answer  8۔ جواب دینے سے انکار کر دیا  9. Don’t know  9۔ نھیں پتہ | ****  ****  ****  **** |
| 2.8 | Did _____ experience pain in the chest in the month preceding death?  کیا ـــــــــــــــــ-کو انتقال سے ایک مہینے پہلے سینے کا درد اٹھا تھا؟ | 1. Yes   1۔ھاں   1. No   2-ناں  8. Refused to answer  8۔ جواب دینے سے انکار کر دیا  9. Don’t know  9۔ نھیں پتہ | ****  ****  ****  **** |
|  | *If “No” or “Don’t know” or “Refused to answer” go to 2.11*  *اگر 'ناں' یا '* نھیں پتہ' تو 11۔2 پہ جائیں | |  |
| 2.10 | How long did the pain last?  درد کتنی دیر کا تھا؟ | 1. Less than 30 minutes   1۔ 30 منٹ سے کم   1. 30 minutes to 24 hours   2۔ 30 منٹ سے 24 گھنٹے   1. More than 24 hours   3۔ 24 گھنٹے سے زیادہ  8. Refused to answer  8۔ جواب دینے سے انکار کر دیا  9. Don’t know  9۔ نھیں پتہ | ****  ****  ****  ****  **** |
| 2.11 | Was there blood in the stool?  کیا پاخانے میں خون آتا تھا؟ | 1. Yes   1۔ھاں   1. No   2-ناں  8. Refused to answer  8۔ جواب دینے سے انکار کر دیا  9. Don’t know  9۔ نھیں پتہ | ****  ****  ****  **** |
|  | *If “No” or “Don’t know” or “Refused to answer” go to 2.13*  *اگر 'ناں' یا '* نھیں پتہ' تو 13۔2 پہ جائیں | |  |
| 2.12 | Was there blood in the stool up until death?  کیا پاخانے خون انتقال کے وقت تک آتا تھا؟ | 1. Yes   1۔ھاں   1. No   2-ناں  8. Refused to answer  8۔ جواب دینے سے انکار کر دیا  9. Don’t know  9۔ نھیں پتہ | ****  ****  ****  **** |
| 2.13 | Did _____ vomit in the week preceding the death?  کیا ـــــــــــــــــــ کوانتقال سے ایک ہفتے پہلے الٹی ہوئی تھی؟ | 1. Yes   1۔ھاں   1. No   2-ناں  8. Refused to answer  8۔ جواب دینے سے انکار کر دیا  9. Don’t know  9۔ نھیں پتہ | ****  ****  ****  **** |
|  | *If “No” or “Don’t know” or “Refused to answer” go to 2.16*  *اگر 'ناں' یا '* نھیں پتہ' تو 16۔2 پہ جائیں | |  |
| 2.14 | Was there blood in the vomit?  کیا الٹی میں خون تھا؟ | 1. Yes   1۔ھاں   1. No   2-ناں  8. Refused to answer  8۔ جواب دینے سے انکار کر دیا  9. Don’t know  9۔ نھیں پتہ | ****  ****  ****  **** |
| 2.15 | Was the vomit black?  کیا الٹی کالے رنگ کی تھی؟ | 1. Yes   1۔ھاں   1. No   2-ناں  8. Refused to answer  8۔ جواب دینے سے انکار کر دیا  9. Don’t know  9۔ نھیں پتہ | ****  ****  ****  **** |
| 2.16 | Did ______ experience a period of loss of consciousness?  کیا ـــــــــــــکچھ وقت کے لیئے بےہوش ہو گئے تھے؟ | 1. Yes   1۔ھاں   1. No   2-ناں  8. Refused to answer  8۔ جواب دینے سے انکار کر دیا  9. Don’t know  9۔ نھیں پتہ | ****  ****  ****  **** |
|  | *If “No” or “Refused to answer” or “Don’t know” thank the participat and end the interview*  *اگر 'ناں' یا '* نھیں پتہ' تو شرکا کا شکریہ ادا کریں اور انٹرویو ختم کر دیں | | |
| 2.17 | Did the period of loss of consciousness start suddenly or slowly?  *کیا* بےہوشی کا دورہ اچانک شروع ہوا تھا یا آہستہ آہستہ؟ | 1. Suddenly   1۔ اچانک   1. Slowly   2۔ آہستہ آہستہ  8. Refused to answer  8۔ جواب دینے سے انکار کر دیا  9. Don’t know  9۔ نھیں پتہ |  |
| 2.18 | Did it continue until death?  کیا بےہوشی انتقال تک جاری تھی؟ | 1. Yes   1۔ھاں   1. No   2-ناں  8. Refused to answer  8۔ جواب دینے سے انکار کر دیا  9. Don’t know  9۔ نھیں پتہ | ****  ****  ****  **** |
